# Nicotine metabolite ratio and attitude towards nicotine metabolite ratio informed smoking cessation care in Jordan

**DOI:** 10.18332/tpc/217141

**Published:** 2026-04-04

**Authors:** Ahmed Salem, Fanar Alsmarat, Tasneem Al-Awaida, Layth Al-Ramahi, Yousef Shawawrah

**Affiliations:** 1 Department of Anatomy, Physiology and Biochemistry, Faculty of Medicine, The Hashemite University, Zarqa, Jordan; 2 Department of Medical Laboratory Science, Faculty of Applied Medical Science, The Hashemite University, Zarqa, Jordan

**Keywords:** smoking, attitude, cotinine, passive smoking, nicotine metabolite ratio

## Abstract

**INTRODUCTION:**

Jordan has one of the highest global smoking and secondhand smoke (SHS) exposure rates. Nicotine metabolite ratio (NMR), the ratio of nicotine metabolites trans-3'-hydroxycotinine to cotinine, serves as a marker for tailored smoking cessation strategies to improve abstinence rates. This study examined smokers' NMR status, attitudes toward NMR-informed smoking cessation care, and SHS exposure markers in non-smokers exposed and not exposed to SHS.

**METHODS:**

This cross-sectional study recruited participants using convenience sampling from Amman and Madaba, Jordan. Participants were categorized into current smokers, non-smokers not exposed to SHS, and non-smokers exposed to SHS. Smokers were classified as slow (NMR <0.31) or fast (NMR ≥0.31) metabolizers. A baseline questionnaire gathered demographics, smoking data, and attitudes toward NMR-informed smoking cessation care. Statistical analyses, including descriptive statistics, chi-squared tests, and non-parametric tests were performed.

**RESULTS:**

A total of 251 participants were recruited: 125 smokers (49.8%), 63 non-smokers exposed to SHS (25.1%), and 63 non-smokers not exposed to SHS (25.1%). Median cotinine levels were high in non-smokers exposed to SHS (36.1 ng/mL) and non-smokers not exposed to SHS (32 ng/mL). Among smokers, 105 (84%) were fast nicotine metabolizers (NMR ≥0.31) and 20 (16%) were slow metabolizers (NMR <0.31), with a median NMR of 0.52 (range: 0.14–6.6). Most smokers showed a positive attitude towards NMR-informed smoking cessation care.

**CONCLUSIONS:**

The predominance of fast nicotine metabolizers in this study underscores the need for NMR-tailored cessation approaches. High SHS exposure observed underscores the need to enforce strict public smoking restrictions in Jordan.

## INTRODUCTION

Tobacco smoking is one of the leading causes of many lethal diseases, including cancer and other non-communicable diseases. Jordan has one of the highest smoking rates in the world, with over 42% of the adult population (1.7 million) actively smoking^1^. Exposure to secondhand smoke (SHS) poses an additional public health challenge in Jordan, with approximately 80% of Jordanians regularly exposed to it^1^.

Standard smoking cessation approaches utilizing FDA-approved drugs such as bupropion, nicotine replacement therapy (NRT), and varenicline can enhance the likelihood of quitting. However, there is only a modest success rate for these treatments, with quit rates typically not exceeding 30%^2^. In Jordan, about 40% of active smokers have attempted to quit but have not succeeded^1^. Although national abstinence rates are not available, studies focused on specific patient groups indicate that these rates have not surpassed 25%^3,4^.

Substantial efforts have been made to promote smoking cessation using established standard guidelines in smoking cessation clinics in Jordan. Despite this, smoking prevalence remains alarmingly high^5^. There is an urgent public health need to understand the underlying reasons behind Jordan’s high smoking prevalence rate^1^. Personalized interventions, tailored to smokers’ genetic predisposition or nicotine dependence, could increase the cessation rate among Jordanian smokers^6^.

The nicotine metabolite ratio (NMR), which reflects the ratio of the nicotine metabolites trans-3'-hydroxycotinine to cotinine, is a genetically informed biomarker that has been proven to predict smokers’ nicotine dependence, likelihood of achieving smoking cessation, and response to various FDA-approved cessation treatments, enabling personalized smoking cessation therapy^6,7^. Smokers are classified as either fast nicotine metabolizers (NMR ≥0.31) or slow nicotine metabolizers (NMR <0.31) based on their NMR values^7^. Categorizing smokers into distinct groups can help healthcare providers customize treatment strategies for better results. However, the current Jordanian smokers’ NMR status and their attitude towards NMR-informed cessation care are unknown. Therefore, this study aimed to investigate Jordanian smokers’ NMR status and their attitude toward NMR-informed smoking cessation care. Additionally, this study assessed cotinine and carbon monoxide levels among non-smokers not exposed to SHS and non-smokers exposed to SHS as markers of SHS exposure.

## METHODS

### Study design

This study employs a cross-sectional design. Recruitment occurred from 14 November 2023 to 2 March 2024, in Amman and Madaba, Jordan. The target sample was divided into three groups based on smoking status: current smokers, non-smokers not exposed to SHS, and non-smokers exposed to SHS. Convenience sampling was used, with the attention that recruitment closely reflects Jordanian age and gender distribution among eligible participants. Eligibility criteria included adults aged 25–64 years. Individuals with a diagnosis of cancer or cardiovascular diseases were excluded from this study. Eligible participants provided blood samples for analysis and completed a baseline questionnaire.

### Baseline demographic and smoking status determination

A baseline questionnaire was used to collect relevant information on demographics and smoking status. The questionnaire contained questions regarding participants’ current and prior smoking status, the Fagerström test for nicotine dependence (FTND) to assess the level of nicotine dependence (for current smokers), and smokers’ attitudes toward the application of NMR-informed smoking cessation care in Jordan. Demographic data such as height, weight, and age were also collected. We ensured a complete collection of data for all participants.

Current smokers were defined as any adult who has smoked 100 cigarettes or more in his or her lifetime and who currently smokes cigarettes every day. Non-smokers not exposed to SHS at home or workplaces were identified as adults who have smoked less than 100 cigarettes in their lifetime and do not live in a home that has another smoker, or work in a place that allows smoking and/or have people smoking regularly inside the workplace. Non-smokers exposed to SHS at home and/or workplaces were defined as adults who have smoked less than 100 cigarettes in their lifetime and are regularly exposed to environmental tobacco smoke from living with a family member who smokes or being in environments where smoking occurs for at least 6 hours daily. Smokers were required to have smoked their last cigarette within the previous 24 hours to ensure accurate measurement of NMR.

To ensure the accuracy of participant classification into non-smokers, exposed to SHS, or current smokers, we utilized a carbon monoxide monitor, using carbon monoxide levels ≥10 ppm as the cutoff value for identifying current smokers. This helped ensure the correct categorization of participants based on carbon monoxide levels, although it was not a requirement for inclusion in any of these groups.

### Blood samples

For nicotine metabolites measurement, a volume of 2 mL of blood was drawn from each participant into an ethylenediaminetetraacetic acid (EDTA)-coated tube (lavender top vacutainers; Samplix^®^/Kremsmünster, Austria). The EDTA tubes underwent immediate centrifugation for 15 min at 4200 rpm to separate plasma. The plasma tubes were stored at -80°C for subsequent analyses.

Analyses were performed using the Thermo Scientific Dionex UltiMate 3000 UHPLC System with LPG-3600 MB Micropump and WPS-3000TBPL Autosampler (Germering, Germany) to quantify cotinine and 3HC levels within blood plasma samples. 3HC was only measured in the plasma samples of current smokers to calculate current smokers’ NMR values.

Samples were prepared by mixing 200 μL of plasma, 200 μL of distilled water, and 200 μL of 10% trichloroacetic acid (TCA). Samples were vortexed for 10 s and then centrifuged at 10000 rpm for 5 min. A volume of 50 μL of the supernatant was injected into the HPLC system with the following conditions: C18 3 μm particle size 1.7×100 mm at 40^o^C at a flow rate of 1.0 mL/min. The total run time was 8 min. Each run had a negative and a positive control. (–)-cotinine solution, 486-56-6, C-016, and (±)-cotinine-d3 solution, 110952-70-0, C-017 (Sigma Aldrich) were used as the standard for cotinine measurement. The trans-3'-hydroxycotinine solution, 34834-67-8, H-101 (Sigma Aldrich) was used as the standard for 3HC measurement. Analyses were performed at Mega Lab medical laboratory, Amman, Jordan.

After cotinine and 3HC levels were measured, we calculated the NMR. Based on this ratio, we classified smokers into slow nicotine metabolizers (NMR <0.31) or fast nicotine metabolizers (NMR ≥0.31).

### Statistical analysis

The primary endpoint of this study is to quantify the NMR in the Jordanian population and assess their attitudes toward metabolite-informed care. Based on previous studies, we anticipated a standard deviation of nicotine metabolism of 0.36. Therefore, we aimed to recruit 125 active smokers, accounting for a power of 80% and an alpha of 0.05. We also assumed a conservative technical failure rate of up to 20%. As this is a pilot study, it may not fully represent the entire population. However, we aimed to recruit a sample that reflects the Jordanian population in terms of age and gender.

Statistical analyses were performed using Statistical Package for Social Sciences (SPSS) version 25 (IBM, USA). Descriptive analyses were used to determine the prevalence of variables. The chi-squared test was used to estimate the correlation between categorical variables. The Kruskal-Wallis test was used to compare more than 2 mean ranks. The Mann-Whitney U test was used for pairwise comparisons of continuous variables between two groups. A significance level of p<0.05 was considered statistically significant.

## RESULTS

### Demographic overview of the study population

A total of 251 participants were recruited for this study. The median age for the study population was 43 years (range: 25–64 years). There were 125 participants (49.8%) in the current smoker group. The non-smokers exposed to SHS and the non-smokers not exposed to SHS groups each comprised 63 participants (25.1%). The median body mass index (BMI) was 29.0 kg/m^2^ (range: 17.1–53.3) (Table 1).

**Table 1:** Demographic characteristics of study participants, Jordan (N=251)

*Characteristics*	*All* *(N=251)*	*Current smokers* *(N=125)*	*Non-smokers exposed to SHS* *(N=63)*	*Non-smokers not exposed to SHS* *(N=63)*	*p*
**Males**, n (%)	135 (53.8)	67 (53.6 )	34 (53.9 )	34 (53.9 )	0.998
**Age** (years), median (range)	43 (25–64)	43 (25–64)	40 (25–64)	44 (25–64)	0.976
**BMI** (kg/m^2^), median (range)	29.0 (17.1–53.3)	27.8 (17.1–48.4)	29.1 (17.4–48.0)	29.9 (18.7–53.3)	0.295

BMI: body mass index.

### Smoking markers among study participants

The median carbon monoxide level across all participants was 13 ppm. Among current smokers, non-smokers exposed to SHS, and non-smokers not exposed to SHS, the median carbon monoxide levels were 18, 5, and 3, respectively (p<0.0001). Cotinine median levels were also significantly higher in current smokers (660 ng/mL) compared to non-smokers exposed to SHS (36.1 ng/mL) and non-smokers not exposed to SHS (32 ng/mL) (p<0.0001). The median level of 3HC in current smokers was 340 ng/ mL (Table 2).

**Table 2:** Smoking indexes and biomarkers among study participants, Jordan (N=251)

*Variable*	*All participants* *(N=251)*	*Current smokers* *(N=125)*	*Non-smokers exposed to SHS* *(N=63)*	*Non-smokers not exposed to SHS* *(N=63)*	*p*
**Carbon monoxide** (ppm), median (range)	13 (1–51)	18 (2–51)	5 (1–38)	3 (1–12)	<0.0001
≥10 ppm, n (%)	116 (46.2)	98 (78.4)	16 (25.4)	1 (1.6)	
**Cotinine** (ng/mL), median (range)	258.0 (2.5–1685.2)	660.0 (22.0–1685.2)	36.1 (2.5–655.3)	32.0 (2.5–670.5)	<0.0001
**3HC** (ng/mL), median (range)		340 (33–1044.1)			

CO: carbon monoxide. 3HC: trans-3'-hydroxycotinine.

The Mann-Whitney U test was conducted to compare cotinine levels between males (n=135, mean rank=125.47, median=211.5, range=2.8–1685.2) and females (n=116, mean rank=126.62, median=298.2, range=2.5–1198.3); the result indicated no significant difference (p=0.901).

### Nicotine metabolite ratio (NMR)

Of the current smokers, 105 (84%) were fast nicotine metabolizers (NMR ≥0.31), while 20 (16%) were slow nicotine metabolizers (NMR <0.31). The median NMR value was 0.52 (0.14–6.6). The median nicotine dependence score among all current smokers was 5 (0–10). The majority of fast nicotine metabolizers 61 (58.1%) were aged ≤44 years, while the majority of slow nicotine metabolizers 12 (60%) were aged >44 years (p=0.017). No significant differences were found in the number of cigarettes smoked per day, FTND score, gender, or smoking duration between fast and slow nicotine metabolizers: p=0.452, 0.453, 0.069, and 0.624, respectively (Table 3).

**Table 3:** Differences in descriptive smoker characteristics by nicotine metabolite ratio among smokers in Jordan

*Variable*	*Fast nicotine metabolization* *(NMR ≥0.31)* *n (%)*	*Slow nicotine metabolization* *(NMR <0.31)* *n (%)*	*p*
**Total**	105 (84.0)	20 (16.0)	
**Cigarettes/day**			0.452
1–10	23 (21.9)	7 (35.0)	
11–20	39 (37.1)	6 (30.0)	
>20	43 (41.0)	7 (35.0)	
**Smoking duration**			0.624
≤10	36 (34.3)	8 (40.0)	
>10	69 (65.7)	12 (60.0)	
**FTND score**			0.453
<5	43 (41.0)	10 (50.0)	
≥5	62 (59.0)	10 (50.0)	
**Gender**			0.069
Males	60 (57.1)	7 (35.0)	
Females	45 (42.9)	13 (65.0)	
**Age** (years)			0.017
25–34	38 (36.2)	1 (5.0)	
35–44	23 (21.9)	7 (35.0)	
45–54	31 (29.5)	10 (50.0)	
55–64	13 (12.4)	2 (10.0)	

FTND: Fagerström test for nicotine dependence.

### Jordanian attitudes toward nicotine metabolites-informed cessation care and smoking behaviors

The majority of participants showed a positive attitude toward NMR-informed smoking cessation care. When asked: ‘Do you agree to do a blood test to determine how your body breaks down nicotine to help you quit smoking, and do you believe it is a good medical advancement that will assist in quitting smoking by matching you with the most appropriate medications?’, 122 (97.6%) agreed, while only 3 (2.4%) disagreed. Additionally, in response to the item: ‘If the NMR blood test indicates a higher likelihood of difficulty quitting smoking, it would deter me from trying to quit or negatively affect my motivation’, 110 (88%) disagreed and 15 (12%) agreed.

Female smokers were found to smoke fewer cigarettes per day, starting later, and for less duration (all p<0.0001) (Table 4).

**Table 4:** Difference in the smoking behaviors between female and male smokers in Jordan, (N=125)

*Variable*	*Females (N=58)* *n (%)*	*Males (N=67)* *n (%)*	*χ^2^/Fisher*	*p*
**Cigarettes/day**			26.540	<0.0001
1–10	25 (43.1)	5 (7.5)		
11–20	21 (36.2)	24 (35.8)		
>20	12 (20.7)	38 (56.7)		
**Years since started smoking**			43.604	<0.0001
≤10	38 (65.5)	6 (9.0)		
>10	20 (34.5)	61 (91.0)		
**Age started smoking** (years)				<0.0001
≤18	47 (81.0)	24 (35.8)	3.312	
>18	11 (19.0)	43 (64.2)		

## DISCUSSION

Smoking poses a major challenge in Jordan, both in terms of health and economic impact. The combined smoking and SHS exposure economic impact in Jordan was estimated to be approximately $2.1 billion, making up 4.7% of Jordan’s gross domestic product^8^. Tobacco smoking accounted for 85% of the total economic cost, while SHS exposure accounted for 15%^8^. The current study investigated Jordanian active smokers’ smoking behavior and nicotine metabolism in addition to assessing smoke exposure among non-smokers exposed to SHS and non-smokers not exposed to SHS. In our analysis, the median NMR value was relatively high (0.52), and the majority of our Jordanian active smokers’ sample (84%) were fast nicotine metabolizers (NMR ≥0.31). Compared to other populations, the proportion of fast nicotine metabolizers and the median NMR value in our sample of Jordanian smokers are significantly higher. The proportion of fast nicotine metabolizers typically ranges from 50% to 70%^9-12^. Fast nicotine metabolizers tend to smoke more frequently, consume more cigarettes, face more intense withdrawal symptoms, and have stronger nicotine dependence, making it harder for them to quit compared to slow nicotine metabolizers^7,13,14^. These findings have major implications for the design of future smoking cessation interventions in Jordan.

The median values of cotinine in this study were 660 ng/mL for current smokers, 36.1 ng/mL for non-smokers exposed to SHS, and 32 ng/mL for non-smokers not exposed to SHS. The levels of cotinine reported in our study align with the results of a previous study that evaluated nicotine and cotinine levels in the Jordanian population, which reported that the average plasma cotinine level in smokers and non-smokers was 379.4 ng/mL and 50 ng/mL, respectively^15^. However, in comparison to other populations, Jordanian smokers and non-smokers have significantly higher levels of cotinine in their plasma^16-21^. Genetic differences in nicotine metabolism and the high smoking intensities among Jordanians could explain this.

Regarding carbon monoxide levels, current smokers had a median carbon monoxide level of 18 ppm, compared to 5 ppm for non-smokers exposed to SHS and 3 ppm for non-smokers not exposed to SHS. However, 25% of participants in the non-smokers exposed to SHS cohort had ≥10 ppm carbon monoxide levels. This could suggest that some participants who identified themselves as non-smokers exposed to SHS might have misreported their smoking status. Additionally, the close similarity of cotinine and carbon monoxide levels between non-smokers exposed to SHS and non-smokers not exposed to SHS could also highlight the unintentional exposure of non-smokers in the non-smokers not exposed to SHS cohort to environmental smoke and suggests that non-smokers are frequently exposed to substantial amounts of tobacco smoke, likely due to the widespread and socially accepted practice of smoking in public and private spaces in Jordan. This practice is in contradiction with the law, where it is prohibited to smoke in public places according to the Public Health Law in Jordan, but this is rarely strictly enforced^22^. This underscores the urgent need for stricter enforcement of smoke-free policies and increased public awareness to reduce SHS exposure.

The number of cigarettes smoked per day, gender, nicotine dependence, and age were among the most reported factors to be significantly correlated with NMR. Typically, females, older individuals, and smokers with higher dependence scores tend to have higher NMR values^23-26^. However, in our study, fast nicotine metabolizers were younger than slow nicotine metabolizers. Additionally, no significant differences were observed between fast and slow nicotine metabolizers in terms of the number of cigarettes smoked per day, gender, nicotine dependence score, or smoking duration. These findings align with previous studies that also failed to demonstrate significant differences in these variables, which may be attributable to the relatively small sample size^11,27-29^. Furthermore, no significant gender differences were observed in cotinine levels. However, gender differences were notable in smoking behaviors; men tended to smoke more cigarettes per day, for a longer duration, and starting much earlier compared to women. Social, cultural, or behavioral factors may have influenced these differences^30^.

The majority of smokers in this study expressed willingness to do a blood analysis to determine their NMR to assess the most appropriate smoking cessation treatment, and most respondents rejected the notion that the result of the NMR would deter them from attempting to quit smoking. Smokers with slow nicotine metabolism (NMR <0.31) respond better to NRT compared to fast nicotine metabolizers. Conversely, fast nicotine metabolizers typically exhibit better responses and experience fewer side effects when treated with varenicline and bupropion^6^. Matching treatment with NMR status (treating slow nicotine metabolizers with the NRT and fast nicotine metabolizers with varenicline) has the potential to reduce side effects and save costs by ensuring that smokers are treated with tailored regimens that can result in greater abstinence rates, lower side effects, and lower likelihood of using certain medications needlessly. The application of NMR-informed smoking cessation care in Jordan has the potential to increase smoking cessation rates among Jordanian smokers significantly. However, future randomized controlled trials are necessary to validate these findings and establish the clinical utility and efficacy of personalized smoking cessation therapies based on NMR values in Jordan.

### Limitations

This study has several limitations. Firstly, the reliance on baseline questionnaires for smoking status verification may have introduced biases or inaccuracies if participants misreported their smoking habits. The study recruited participants from specific regions in Jordan (Amman and Madaba), which may not represent the broader Jordanian population. Additionally, the sample size of 251 participants, although adequate for the study’s objectives, may not be sufficient to capture the diversity of smoking behaviors and nicotine metabolism patterns across different demographic groups within Jordan, for which a larger random sample would be needed.

## CONCLUSIONS

Given the high prevalence of fast nicotine metabolizers and positive attitude towards NMR-metabolite-informed smoking cessation care in our sample of the Jordanian population, tailoring treatments based on an individual’s NMR status could improve quit rates and reduce the likelihood of relapse while reducing treatment side effects and limiting unnecessary costs. However, further randomized controlled trials are needed to validate these findings and establish the efficacy of personalized smoking cessation therapies based on NMR values in Jordan. The study’s findings also highlight the alarming levels of SHS exposure. Non-smokers exposed to SHS participants exhibited significantly high carbon monoxide and cotinine levels. Furthermore, even though they may not fully recognize it, non-smokers not exposed to SHS in this study were found to be impacted by SHS at levels similar to non-smokers exposed to the SHS cohort, underscoring the need for more stringent enforcement of smoking restrictions in public areas.

## Data Availability

The data supporting this research are available from the authors on reasonable request.
